# Prophylactic folinic acid prevents pemetrexed myelosuppression: A randomized trial toward safer treatment with chemotherapy in non-small cell lung cancer

**DOI:** 10.1016/j.neo.2026.101343

**Published:** 2026-08-01

**Authors:** Ramon Contrucci, Rob ter Heine, Merel Tonn, Jeroen Diepstraten, Eric van Thiel, Charlotte van Kesteren, Michel van den Heuvel, Cor van der Leest, Nikki de Rouw

**Affiliations:** a Department of Pharmacy, Pharmacology & Toxicology, Radboudumc, Research Institute for Medical Innovation, Nijmegen, The Netherlands; b Department Clinical Pharmacy, Amphia Hospital, Breda, The Netherlands; c Department Clinical Pharmacy, Dijklander Hospital, Hoorn, The Netherlands; d Department of Pulmonary Diseases, Albert Schweitzer Hospital, Dordrecht, The Netherlands; e Department Clinical Pharmacy, Albert Schweitzer Ziekenhuis, Dordrecht, The Netherlands; f Department of Pulmonary Diseases, Radboudumc, Research Institute for Medical Innovation, Nijmegen, The Netherlands; g Department of Pulmonary Diseases, University Medical Center Utrecht, Utrecht, The Netherlands; h Department of Pulmonary Diseases, Amphia Hospital, Breda, The Netherlands

**Keywords:** NSCLC, Folinic acid, Pemetrexed, Myelosuppression, Neutropenia, Toxicity

## Abstract

Background Pemetrexed is a cornerstone in advanced non-small cell lung cancer treatment. Although generally well tolerated, severe myelosuppression occurs in 26% of patients. Preventing chemotherapy-associated toxicity has become increasingly important with the introduction of osimertinib combined with pemetrexed-based chemotherapy. Due to substantial toxicity, this regimen is often not administered to frail patients. Folinic acid prophylaxis can mitigate pemetrexed-induced toxicity, however its preventive use has not been routinely studied. We aimed to investigate the efficacy of folinic acid prophylaxis to reduce myelosuppression. Methods Fifty patients treated with pemetrexed were randomized (1:1) to receive pemetrexed with or without oral folinic acid prophylaxis on days 2–4 after each chemotherapy cycle. The primary endpoint was absolute neutrophil count (ANC) after the first chemotherapy cycle. Secondary endpoints included ANC after the second cycle, grade neutropenia, treatment efficacy, renal function and incidence of dose modifications. Results Twenty-four patients received folinic acid and twenty-six served as controls. Higher ANC were observed in the folinic acid group after the first cycle (median 3.79; IQR 2.22–4.93 vs. 1.85; IQR 1.43–3.78; p

## Introduction

Pemetrexed remains a cytotoxic fundament in the treatment of non-small cell lung cancer (NSCLC). Combined with immunotherapy pemetrexed forms the backbone of chemoimmunotherapy, which has revolutionized the treatment landscape of NSCLC [[2], [3], [4], [5]]. Although effective, pemetrexed is associated with serious toxicity [6,7]. In combination with immunotherapy or targeted therapies, the cumulative toxicity of these agents is cause for even more precaution [8,9]. Also, the advancements in oncological treatment have improved overall survival rates [2]. Consequently, treatment duration increases, and patients are becoming older and more susceptible to treatment-related toxicity [10,11]. Careful management of pemetrexed-related toxicity is essential and even more crucial with the combination of chemotherapy with immunotherapy or targeted therapy to ensure both effective and safe treatment [7].

Pemetrexed is associated with myelosuppression as a treatment-limiting toxicity. Despite vitamin B12 and folic acid, which are already standard of care to prevent pemetrexed toxicity, up to 26% of patients experience severe (grade III or IV) neutropenia [6,7,[13], [14], [15]]. Pemetrexed in itself is generally a well-tolerated chemotherapy agent. However, the cumulative effect with other chemotherapy agents still results in high incidences of toxicity. The incidence of neutropenia in patients receiving chemoimmunotherapy may even be higher, as neutropenia is associated with creatinine clearance and chemoimmunotherapy is known to cause renal toxicity [2,8,16,17]. Furthermore, since the advent of targeted therapy with osimertinib combined with pemetrexed-based chemotherapy (FLAURA2 regimen), treatment tolerability has become a major issue. In this combination therapy 64% of the study population experienced a grade III or higher adverse event, limiting the applicability of this treatment in patients with poor performance [4,5]. Incidence of any grade hematological toxicity was 48% [5]. Myelosuppression is directly associated with an increased risk of infections, which consequently decreases quality of life and can result in hospitalization or even death. It is also a major cause of delays or cessation of oncological treatment [7,10,11]. Moreover, pemetrexed-induced toxicity also has a substantial economic impact [18]. There is, therefore, an urgent need to improve the safety profile of pemetrexed.

A promising strategy to prevent pemetrexed-induced neutropenia is the prophylactic use of folinic acid [7]. Folinic acid is the activated form of folic acid. Unlike folic acid, folinic acid can directly mitigate pemetrexed-induced toxicity [7,[19], [20], [21]]. Preclinical research has already demonstrated the potential of folinic acid to mitigate pemetrexed-induced toxicity [[19], [20], [21]]. We have previously shown that the prophylactic administration of folinic acid enables safe and effective treatment with pemetrexed in patients with impaired renal function[22]. Furthermore, the drug label of pemetrexed already recommends high-dose intravenous folinic acid for the treatment of severe neutropenia[7,[23], [24], [25]]. Oral folinic acid is also approved for preventing hematological toxicity induced by methotrexate. Since both methotrexate and pemetrexed are antifolates, and the hematological toxicity caused by both agents appears to be primarily driven by inhibition of the enzyme dihydrofolate reductase (DHFR), we argue that folinic acid can similarly prevent hematological toxicity caused by pemetrexed[7].

We, therefore, aimed to evaluate the effect of oral prophylactic folinic acid on neutropenia in patients treated with pemetrexed.

## Methods

### Study design

We performed a randomized, open-label, double-arm study (FLEX-trial) in two large teaching hospitals (Amphia Hospital and Albert Schweitzer Hospital) in the Netherlands. The Medical Ethics Committee (METC) Oost-Nederland (Nijmegen, the Netherlands) approved the study protocol. This trial was conducted according to the protocol compliant with Good Clinical Practice, and the Declaration of Helsinki. This trial was registered in ClinicalTrials.gov, number NCT06010277 and the European Clinical Trial Registration System (CTIS) EU-CT 2022-502345-10-00.

### Participants

Eligible patients were aged 18 years or older, diagnosed with stage IV NSCLC treated with pemetrexed (500mg/m2) combined with platinum induction chemotherapy, and had a Cooperative Oncology Group performance status (ECOG PS) of 0, 1 or 2. Exclusion criteria were contraindications or drug-drug interactions with folinic acid according to the Summary of Product Characteristics (SmPC)[26], previous hypersensitivity reactions to folinic acid or any of the excipients, or anemia caused by vitamin B12 deficiency. Patient recruitment and eligibility assessment were the responsibility of the treating physician. All patients provided written informed consent.

### Randomization

Participants were randomly assigned in a 1:1 ratio in either a folinic acid prophylaxis group or a control group. Patients were randomized by a qualified research professional using a validated and secure web-based system (Castor EDC). Randomization was stratified by chemotherapy regimen and the use of proton pump inhibitors (PPIs) since the use of PPIs is associated with increased pemetrexed toxicity[27,28].

### Study procedures

The folinic acid group received standard of care plus oral folinic acid (Rescuvolin). Folinic acid was dosed 45 mg, four times daily and was started 24 hours after the administration of pemetrexed. Folinic acid was continued for 3 days after each cycle of chemotherapy. The control group received standard care without folinic acid. All patients received standard toxicity prophylaxis with folic acid, 0.5mg once daily. The study period consisted of four cycles of chemotherapy.

The dose of folinic acid, 45mg four times daily, was based on comparable doses of folinic acid for the mitigation of the toxic effect of methotrexate, a more potent inhibitor of DHFR compared to pemetrexed[29]. The 24-hour time interval between the administration of pemetrexed and the start of folinic acid prophylaxis enabled efficient polyglutamation of pemetrexed in malignant cells and thus ensured the efficacy of oncological treatment[[29], [30], [31]]. The duration of folinic acid prophylaxis for 3 days was based on a pharmacokinetic/pharmacodynamic analysis. This analysis demonstrated that patients with a creatinine clearance of 45ml/min (which is the lowest acceptable renal function according to the drug label of pemetrexed) are most susceptible to pemetrexed-induced toxicity and maintain toxic pemetrexed plasma concentrations up to 84 hours after administration of pemetrexed[16,25]. Folinic acid prophylaxis duration was based on this worst-case scenario of 84 hours above the toxicity threshold.

Standard-of-care blood samples to determine the absolute neutrophil count (ANC), hemoglobin and renal function were collected before each cycle of chemotherapy. Additional blood samples were collected between days 8 to 10 after the administration (nadir) of the first and second cycles of chemotherapy. Response CT scans were made after the second and fourth cycles of chemotherapy as a part of standard care.

### Outcomes

The primary endpoint was the difference in ANC between the folinic acid group and the control group, 8 to 10 days after the first cycle of chemotherapy. ANC is a surrogate endpoint and its clinical relevance rests on a well-characterized, quantitative relationship between the depth and duration of neutropenia and the risk of infection[[32], [33], [34]]. ANC is a direct quantitative measure of pemetrexed dose-limiting toxicity and the conventional pharmacodynamic endpoint for chemotherapy-induced myelosuppression [35,36]. For pemetrexed specifically, the folate-status–neutropenia axis is supported by drug-specific data: pretreatment homocysteine predicts severe neutropenia together with associated grade 3/4 infection, and pemetrexed systemic exposure predicts hematological toxicity and the ANC-time course[1,10,16,36]. Secondary endpoints included the difference in ANC between both groups, 8 to 10 days after the second cycle of chemotherapy and the grade neutropenia according to the Common Terminology Criteria for Adverse Events v5.0 (CTCAE)[12] 8 to 10 days after the first and second cycle of chemotherapy Exploratory endpoints were treatment response after the second and fourth cycle of chemotherapy categorized as response, partial response and progression. Response assessment was locally and unblinded using the RECIST (1.1) criteria. Further exploratory endpoints were: renal function and hemoglobin before every cycle of chemotherapy; reported adverse events of folinic acid, hospital admissions and the incidence of dose reductions and delay or discontinuation of chemotherapy treatment. All outcomes for both study sites were centrally assessed and analyzed in the same laboratory.

### Statistical analysis

For the sample size calculation, an existing pharmacokinetic/pharmacodynamic model for pemetrexed was used for a Monte Carlo simulation, assuming 100% inhibition of the cytotoxic effect in the bone marrow during folinic acid treatment[16]. This represents an idealized best-case scenario that might overestimate the real-world magnitude of the effect of folinic acid. Then a power analysis was performed using a two-sample t-test. Considering that the estimation was based on optimal conditions, we found that 50 patients should be included to obtain a power of at least 0.80 with an alpha of 0.05.

Patients were only included in the analysis when they reached the primary endpoint (per protocol approach). Analysis was performed using IBM SPSS Statistics 29. Baseline characteristics were analyzed using descriptive statistics reported as median and interquartile range. The neutrophil counts, renal function and hemoglobin were analyzed using a Mann-Whitney-U test (95% confidence interval and p-value). A Chi-squared test (% and p-value) was used to analyze the incidence of grade neutropenia, the oncological treatment response and the incidence of dose reductions, delay or discontinuation of chemotherapy.

### Role of the funding source

The funder of the study had no role in study design, data collection, data analysis, data interpretation, or writing of the report.

## Results

**Fig. 1:**
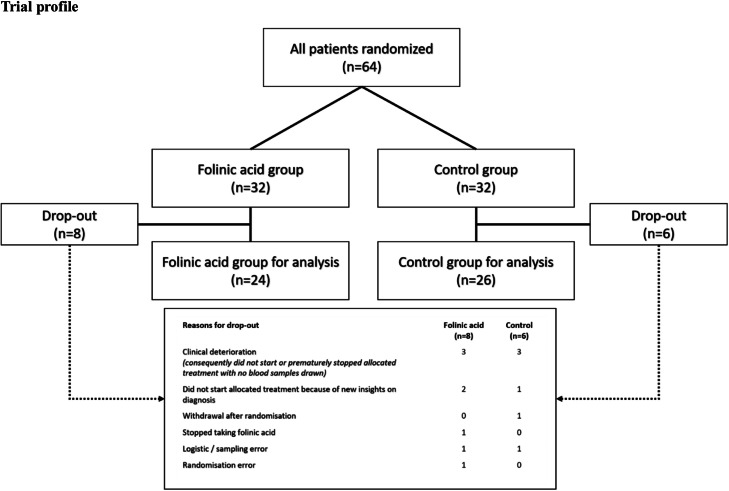
Profile of all patients who were included, enrolled, and dropped out.

In total, 64 patients were included between the 7^th^ of February 2023 and the 28^th^ of June 2024. Fourteen patients did not reach the primary endpoint and dropped out. Eventually, 50 patients enrolled in the study, specifically, 24 patients in the folinic acid group and 26 patients in the control group. Fig. 1 shows a detailed profile of all included patients and trial drop-out per study arm.

Patient characteristics were distributed evenly in both groups. In the folinic acid group, 5 patients had increased homocysteine plasma levels at baseline (>16 mmol/L), compared to 8 patients in the control group, indicating a decreased folate state[1]. Concomitant chemotherapy (carboplatin or cisplatin) or immunotherapy (pembrolizumab) was distributed evenly in both groups. Detailed baseline characteristics are described in Table 1*.*

**Table 1:** Patient characteristics at baseline. kg = kilograms; cm = centimetres; μmol/L = millimolar per litre; ml/min = millilitre per minute; ref. value homocysteine: 4.0 - 16.0 mmol/L^1^; reference neutrophils: No neutropenia >2.0×10^9^ [12].

Patient characteristics	Folinic acid group (n = 24)	control group (n = 26)
Gender (% female)	11 (46)	15 (58)
Age (years)*	71 (65 - 73)	72 (67 - 74)
Weight (kg)*	70 (65 - 84)	68 (63 - 77)
Length (cm)*	171 (166 – 175)	167 (165 - 172)
Diagnose (% NSCLC)	24 (100)	26 (100)
Pemetrexed dose (mg/m^2^)	500 (500-500)	500 (500-500)
Homocysteine (μmol/l)*	12.3 (10.8 – 16.6)	14.7 (11.5 – 18.5)
Neutrophil count baseline, (x10^9^/L) *	5.7 (5.3 – 7.6)	6.1 (5.6 – 8.2)
Estimated glomerular filtration rate, (ml/min) *	89 (78 – 90^#^)	85 (74 – 90^#^)
Chemotherapy regime (%)		
*pemetrexed + carboplatin + pembrolizumab*	13 (54)	14 (54)
*pemetrexed + carboplatin*	8 (33)	9 (35)
*pemetrexed + cisplatin + pembrolizumab*	0 (0)	1 (4)
*pemetrexed + cisplatin*	3 (13)	2 (8)
Proton pump inhibitor (% used)	12 (50)	13 (50)

*Median (95% IQR; Q1 – Q3));

^#^The upper limit cut-off value for estimated glomerular filtration rate was 90ml/min.

**Fig. 2:**
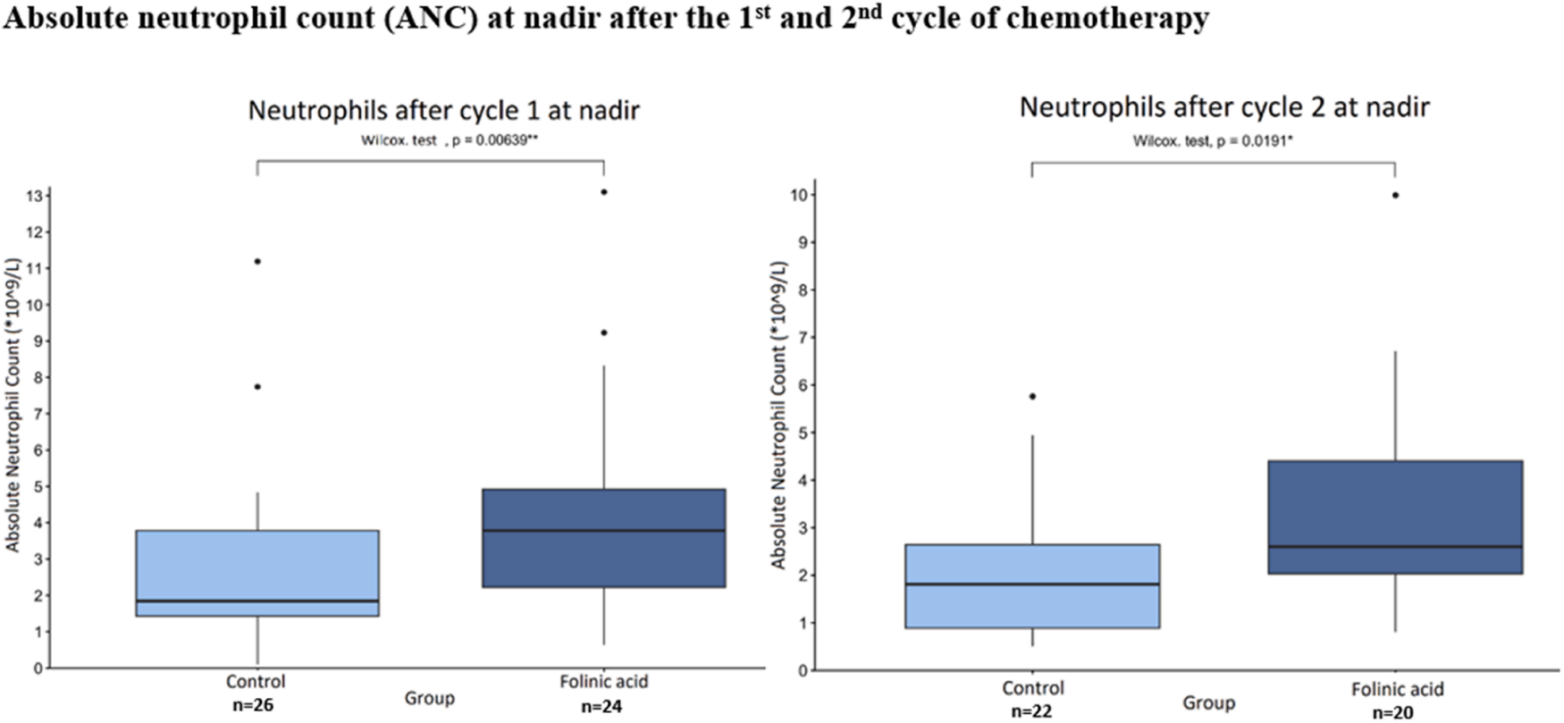
Boxplots displaying the absolute neutrophil count (ANC) after the first and the second cycle of chemotherapy at nadir. The number of patients contributing data after the second cycle of chemotherapy is lower than after the first cycle because 4 patients in the folinic acid group and 4 patients in the control group discontinued treatment after the first cycle of chemotherapy (folinic acid, n = 20; control, n = 22 at cycle 2). Reasons for discontinuation per study arm are reported in the Results section and in table S1 of the supplemental material.

The primary endpoint, ANC (*10^9^) at nadir after the first cycle of chemotherapy, was higher (p < 0.01) in the folinic acid group (median 3.79; IQR 2.22 – 4.93) compared to the control group (median 1.85; IQR 1.43 – 3.78). After the first cycle of chemotherapy, 8 patients discontinued oncological treatment, 4 in the folinic acid group and 4 in the control group. ANC at nadir after the second cycle of chemotherapy were therefore available for 20 patients in the folinic acid group and 22 patients in the control group. After the second cycle of chemotherapy, the ANC (*10^9^) remained higher (p = 0.02) in the folinic acid group (median 2.60; IQR 2.03 – 4.41) compared to the control group (median: 1.76; IQR 0.87 – 2.73). These results are also presented as boxplots in Fig. 2.

After the first cycle of chemotherapy, the incidence of grade I (p = 0.04) and grade II (p = 0.05) neutropenia at nadir was lower in the folinic acid group (Grade I: 4%, Grade II: 0%) compared to the control group (Grade I: 27%, Grade II: 15%). No significant difference was observed for grade III (p = 0.61) or grade IV (p = 0.34) neutropenia. After the second cycle of chemotherapy, no significant difference was observed for grade I ( p = 0.49), grade II (p = 0.74) or grade III (p = 0.08). Grade III neutropenia after the second cycle of chemotherapy occurred in 5% of patients in the folinic acid group compared with 29% of patients in the control group. No grade IV neutropenia was observed after the second cycle of chemotherapy. See table 2 for the detailed results.

**Table 2:** The incidence of grade neutropenia according to the Common Terminology Criteria for Adverse Events v5 after the first and second cycles of chemotherapy in the folinic acid group and the control group. Grade I: ANC 1.5 – 2.0*10^9^/L; Grade II: ANC 1.0 – 1.5*10^9^/L; Grade III: ANC 0.5 – 1.0*10^9^/L; Grade IV: ANC <0.5*10^9^/L. ANC = absolute neutrophil count.

Grade neutropenia	Cycle 1 at nadir			Cycle 2 at nadir		
	Folinic acid group (%)	Control group (%)	p value	Folinic acid group (%)	Control group (%)	p value
Grade I	4%	27%	0.04	11%	19%	0.49
Grade II	0%	15%	0.05	11%	14%	0.74
Grade III	4%	8%	0.61	5%	29%	0.08
Grade IV	0%	4%	0.34	0%	0%	-

No significant difference was observed in the decline of haemoglobin between the first and the fourth cycle of chemotherapy (p = 0.32) between the folinic acid group (median: 1.8; IQR 1.4 – 2.6) compared to the control group (median: 2.0; IQR 1.2 – 2.5). Also, no significant difference was observed in the decline of renal function from the first to the fourth cycles of chemotherapy between both groups (p = 0.57). No significant difference was observed in response to oncological treatment based on the CT scans between both groups after the second (p = 0.84) and fourth (p = 0.52) cycle of chemotherapy. Moreover, no significant difference was observed in hospital admission between both groups 23 admissions in the folinic acid group against 18 admissions in the control group (p = 0.18) Finally, no significant difference was observed between both groups in dose delay (p = 0.65) or cessation (p = 0.42) of oncological treatment. As a result of folinic acid, two patients experienced grade I nausea and one patient grade II nausea which also discontinued folinic acid. No other folinic-acid-related adverse effects were reported. More detailed information on the secondary outcomes can be found in the supplementals (S1-S4).

## Discussion

We demonstrated that oral folinic acid prophylaxis prevents the decrease of the ANC and reduces the incidence of grade I/II neutropenia. The ANC at nadir was significantly higher in patients receiving oral folinic acid after both the first (p = < 0.01) and second (p = 0.02) cycle of chemotherapy. Additionally, less grade I and II neutropenia was observed after the first cycle of chemotherapy. No difference was found for grade III and IV neutropenia. These findings are of importance, considering the major role of pemetrexed as backbone for both immunotherapy and EGFR inhibition in the current treatment landscape, where pemetrexed-toxicity may impair access to effective treatment [32,33].

The oncological response based on the CT-scan after the 2^nd^ and 4^th^ cycle of chemotherapy was exploratively evaluated. No significant difference in oncological response was observed between both groups. However, no conclusions can be drawn based on these findings since our study was not powered for this secondary endpoint.

The primary endpoint, ANC, is a surrogate endpoint for the incidence of infection risk and, further downstream could possibly translate to hospitalizations, febrile neutropenia and death. However, the actual translation of ANC at nadir to these clinically relevant endpoints requires an adequately powered trial, specifically focusing on these endpoints. This proof-of-concept trial provides a rationale for further research to examine these endpoints. In this trial hospitalization and dose delay or cessation of treatment were exploratory assessed as secondary endpoints, but our study was underpowered to demonstrate a significant difference[37,38].

After the second cycle of chemotherapy, grade III neutropenia occurred in 5% of patients in the folinic acid group compared with 29% of patients in the control group (p = 0.08), indicating a trend. This is consistent with the direction of the protective effect seen for low-grade neutropenia after the first cycle. The trial was powered for the primary endpoint only and based on a sample size calculation that assumed complete inhibition of pemetrexed-induced myelosuppression by folinic acid[16]. This was an idealized assumption derived from PK/PD modelling and has not been validated in human studies; a more conservative, validated estimate would have required a substantially larger cohort to detect an effect on severe neutropenia. Adequately powered follow-up trials could therefore potentially demonstrate the protective effects of folinic acid on grade III or higher neutropenia.

A limitation of our study was the follow-up time of 4 cycles of chemotherapy (approximately 3 months) which restricts any conclusions about the durability of folinic acid prophylaxis or cumulative myelotoxicity during extended pemetrexed maintenance treatment. The follow-up period was also arguably too short to detect a significant difference in the secondary endpoints creatinine clearance or hemoglobin between both groups. Especially since pemetrexed is known to cause renal toxicity and anemia after a longer treatment period[11,39]. Another limitation was the open label trial design. Although the primary endpoint was based on the ANC, an objective laboratory parameter unaffected by observer expectation, the grading and attribution of clinical adverse events were not blinded, as well as the assessment the clinical response based on the CT-scans.

In total, 14 patients dropped out before reaching the primary endpoint and were, according to the study protocol, consequently excluded. Dropout was reasonably balanced between both arms and was predominantly driven by clinical deterioration before treatment could be started or the primary endpoint assessed, rather than by treatment allocation itself; the remaining exclusions were mainly attributable to patients who did not initiate the allocated treatment (most often because of a later-diagnosed targetable mutation) and to logistic or sampling issues. Dropout of this nature can introduce selection bias; however, given the small absolute number of dropouts, our study was not powered to formally test whether dropout was systematically related to pemetrexed- or folinic-acid-induced toxicity in either arm. To assess the robustness of the primary endpoint to this dropout, a delta-adjusted tipping-point sensitivity analysis was performed, in which missing nadir ANC values were multiply imputed under a range of penalties applied to the folinic acid arm (supplemental material S5). This analysis also serves as an intention-to-treat-like scenario simulation, allowing external readers to compare our findings with intention-to-treat estimates from other trials.

The timing of folinic acid prophylaxis is essential to ensure pemetrexed efficacy. Based on the extensive polyglutamation, the clearance and toxicity threshold of pemetrexed, folinic acid should be administered at least 24 hours (but not much longer) after pemetrexed administration, to allow anticancer effects [16,30]. The median time above toxicity threshold after pemetrexed administration is 27 hours for patients with adequate creatinine clearance (>90ml/min)[16]. Patients with lower creatinine clearance (45-90ml/min) maintain toxic pemetrexed levels up to 84 hours after administration[16]. These patients arguably benefit more from folinic acid prophylaxis compared to patients with adequate creatinine clearance. In our study however, most patients had a relatively high creatinine clearance. We here draw a parallel with folinic acid prophylaxis of the antifolate drug methotrexate, where folinic acid is safely and effectively administered, 24 hours after administration of methotrexate[7]. Since no subgroup analyses by renal function were prespecified or performed, the magnitude of the protective effect of folinic acid in patients with a lower creatinine clearance remains unknown and should be addressed through stratified enrolment in a dedicated follow-up trial.

The non-steroidal anti-inflammatory drugs (NSAIDs) are also associated with increased toxicity of pemetrexed and were contraindicated for all participants[40]. Participants were informed of this contraindication and reminded by their treating physician as part of standard clinical care throughout the treatment period, consistent with routine management of pemetrexed-related drug interactions. The absence of formal medication diaries precludes quantitative assessment of NSAID exposure as a confounding variable, and recommend structured adherence tracking for future analogous trials.

Strengths of this study were the prospective, multicenter and randomized set-up, as well as the consideration of concomitant medication. All participants were stratified during randomization for the use of proton pump inhibitors, which are associated with increased toxicity of pemetrexed[28]. Finally, a strength was the measurement of homocysteine levels at baseline. Homocysteine is a prognostic biomarker for increased risk of pemetrexed toxicity[1]. We found no statistical difference in homocysteine levels at baseline, indicating that results are not confounded by an increased *a priori* risk of pemetrexed toxicity.

Oral folinic acid prophylaxis offers an additional strategy alongside, for example, granulocyte colony-stimulating factor (G-CSF) prophylaxis: both target different parts of the same clinical problem and are best regarded as complementary rather than competing. Folinic acid acts upstream on the antifolate mechanism itself, is taken orally at home, and is inexpensive and well tolerated. G-CSF acts downstream on granulopoiesis and is used as prophylaxis for patients at high risk of febrile or grade III to IV neutropenia, at the cost of subcutaneous administration, bone pain and considerable expense[41,42]. On the present evidence, folinic acid may be most useful in reducing the burden of mild myelosuppression; further research could demonstrate broader applications in patients at high risk of severe neutropenia..

In conclusion, this study demonstrated that prophylaxis with oral folinic acid is effective in preventing pemetrexed-associated neutrophil reduction and grade I/II neutropenia and indicating a trend for grade III neutropenia.. Our results support the use of folinic acid prophylaxis to prevent pemetrexed-induced neutropenia and provides a rationale for future research to further examine clinically relevant endpoints like quality of life of death. Our findings are particularly relevant in the current therapeutic landscape, where pemetrexed is increasingly used in combination regimens with targeted therapies such as osimertinib and immunotherapy. The combination of pemetrexed with osimertinib has demonstrated efficacy in EGFR-mutated non-small cell lung cancer, but is associated with a high incidence of toxicity that can limit treatment access, particularly in patients with poor performance status [4,5]. Similarly, in immunotherapy-based combination regimens, pemetrexed-related toxicity may restrict treatment application and may, thus, compromise therapeutic outcomes. By mitigating neutropenia and associated complications, folinic acid prophylaxis may enable broader patient access to these effective combination therapies and improve treatment tolerability.

## CRediT authorship contribution statement

**Ramon Contrucci:** Writing – original draft, Visualization, Project administration, Methodology, Investigation, Formal analysis, Data curation, Conceptualization. **Rob ter Heine:** Writing – review & editing, Supervision, Resources, Methodology, Conceptualization. **Merel Tonn:** Writing – review & editing, Visualization, Methodology, Data curation. **Jeroen Diepstraten:** Writing – review & editing, Resources. **Eric van Thiel:** Writing – review & editing, Resources. **Charlotte van Kesteren:** Writing – review & editing, Resources. **Michel van den Heuvel:** Writing – review & editing, Supervision. **Cor van der Leest:** Writing – review & editing, Supervision, Resources, Investigation, Conceptualization. **Nikki de Rouw:** Writing – review & editing, Validation, Supervision, Resources, Methodology, Investigation, Conceptualization.

## Declaration of competing interest

The authors declare that they have no known competing financial interests or personal relationships that could have appeared to influence the work reported in this paper.

## Appendix Supplementary materials

Image, application 1

Image, application 2
