# Supplementary material for: Prophylactic folinic acid prevents pemetrexed myelosuppression: A randomized trial toward safer treatment with chemotherapy in non-small cell lung cancer
Source: Neoplasia. 2026 Aug 1;80:101343. doi: 10.1016/j.neo.2026.101343 (PMC13453476; doi:10.1016/j.neo.2026.101343)
Supplement: Supplementary file 2 [file mmc1.docx]

**Supplementals**

Oral folinic acid prophylaxis prevents pemetrexed-induced neutropenia in patients with adequate renal function: results from a randomised clinical trial

**Discontinuation of oncological treatment**

**Table S1**: Detailed results for discontinuation of oncological treatment for any reason in both the folinic acid group and the control group.

| Cycles of chemotherapy | Folinic acid group (%) | Control group (%) | p-value |
| --- | --- | --- | --- |
| Stopped after cycle 1 | 17% | 15% | 0.91 |
| Stopped after cycle 2 | 21% | 15% | 0.83 |
| Stopped after cycle 3 | 17% | 15% | 0.91 |
| Finished all 4 cycles | 46% | 54% | 0.42 |

| Oncological response | CT-scan after 2 cycles | | | CT-scan after 4 cycles | | |
| --- | --- | --- | --- | --- | --- | --- |
|  | Folinic acid group (%) | Control group (%) | p value | Folinic acid group (%) | Control group (%) | p-value |
| Progression | 21% | 14% | 0.57 | 31% | 16% | 0.41 |
| Stable decease | 58% | 68% | 0.68 | 38% | 38% | 1.00 |
| Remission | 21% | 18% | 0.84 | 31% | 46% | 0.53 |

**Oncological response after the second and fourth cycle of chemotherapy**

**Table S2:** Detailed results for oncological response based on the CT scan after the second and fourth cycles of chemotherapy. The response was determined by the physician and categorised as progression, stable disease or remission.

| Haemoglobin |  | Cycle of chemotherapy | | | |
| --- | --- | --- | --- | --- | --- |
|  |  | Cycle 1  (mmol/L) | Cycle 2 (mmol/L) | Cycle 3 (mmol/L) | Cycle 4 (mmol/L) |
| Folinic acid group | Median:  IQR: | 8,3  7.4 – 8.7 | 7.9  7.4 – 8.2 | 7.3  6.7 – 7.7 | 6.7  6.1 – 7.1 |
| Control group | Median:  IQR: | 8,2  7.6 – 9.4 | 7.1  6.7 – 8.4 | 6.6  6.3 – 7.6 | 6.6  6.2 – 7.4 |

**Haemoglobin before each cycle of chemotherapy**

**Table S3**: Detailed results for haemoglobin measured before each cycle of chemotherapy.

| eGFR |  | Cycle of chemotherapy | | | |
| --- | --- | --- | --- | --- | --- |
|  |  | Cycle 1  (ml/min) | Cycle 2  (ml/min) | Cycle 3 (ml/min) | Cycle 4 (ml/min) |
| Folinic acid group | Median:  IQR: | 89  82 – 90* | 90*  80 – 90 | 90*  84 – 90* | 90*  76 – 90* |
| Control group | Median:  IQR: | 85  74 – 90* | 78  75 – 90* | 76  73 – 90* | 83  79 – 90* |

**Estimated glomerular filtration rate before each cycle of chemotherapy**

**Table S4**: Detailed results for estimated glomerular filtration rate (eGFR) calculated with the Cockcroft Gault, measured before each cycle of chemotherapy. * Measurement >90ml/min were reported as 90ml/min.

**S5 tipping point analysis**

**Rationale**

The primary analysis in this trial is a per-protocol analysis, prespecified in the trial protocol. Fourteen of the 64 randomized patients (22%) did not reach the primary endpoint and were excluded under this approach, a dropout rate high enough to raise the possibility of selection bias: per-protocol analyses can overestimate a treatment effect when exclusion is not independent of prognosis, which an intention-to-treat (ITT) analysis is designed to guard against. An ITT analysis with imputation of the missing primary endpoint was therefore considered as an alternative.

The excluded patients do not have a valid ANC nadir available, so an ITT analysis would necessarily rest on imputing the primary endpoint itself rather than a secondary or partially observed variable. For patients whose treatment was stopped or never started because of clinical deterioration, the same deterioration that prevented endpoint assessment plausibly also affected their underlying hematological status, which makes a missing-at-random (MAR) assumption difficult to defend for this subgroup. A single-imputation ITT analysis resting on an assumption that cannot be defended would risk introducing more distortion than it resolves in a trial of this size, particularly given the size of the excluded group.

The per-protocol analysis was therefore retained as primary, and a delta-adjusted pattern-mixture (tipping-point) sensitivity analysis was performed instead. This approach tests whether the 22% dropout rate could plausibly have overturned the primary conclusion through non-random missingness, without requiring MAR to hold: it estimates the magnitude of departure from MAR that would be needed to reverse the result, providing an ITT-like scenario for comparison with other trials without requiring the missing endpoint itself to be observed.

**Methods**

Missing nadir ANC values for the fourteen excluded patients were multiply imputed, anchored at MAR (i.e., dropouts were assumed to resemble completers of their own arm). A departure parameter δ (in ×10⁹/L) was then introduced: across 4000 stochastic imputations per value of δ, progressively less favorable ANC nadir values were assigned to the folinic acid dropouts and progressively more favorable values to the control dropouts, biasing the comparison maximally against the observed treatment effect. The primary Mann-Whitney-U comparison was re-estimated on the full randomized population (n = 64) at each value of δ. This was assessed under two scenarios: both arms stressed simultaneously, and only the folinic acid arm stressed (control dropouts held at their MAR-based values). The tipping point was defined as the smallest value of δ at which the between-group difference in ANC at nadir was no longer statistically significant (p ≥ 0.05). Analyses were performed in RStudio 2025.09.1+401 "Cucumberleaf Sunflower".

**Results and interpretation**

Under MAR (δ = 0), the result was robust: over 99% of imputed datasets remained significant, with a median p-value of approximately 0.002, consistent with the per-protocol result (p < 0.05). When both arms were stressed simultaneously, significance was lost only beyond δ ≈ 1.1 ×10⁹/L, at which point the imputed folinic acid dropouts (median nadir ≈ 2.5 ×10⁹/L) would need to fare worse than the imputed control dropouts (median nadir ≈ 3.1 ×10⁹/L) — that is, the direction of the treatment effect would need to reverse among the roughly 22% of patients who dropped out. When only the folinic acid arm was stressed, significance was threatened only beyond δ ≈ 2.1 ×10⁹/L, corresponding to the assumption that the folinic acid dropouts derived essentially no prophylactic benefit at all, with their nadirs pulled down to control-group levels.

Both tipping points therefore require assumptions that contradict both the observed data and the clinical rationale for folinic acid prophylaxis, and the documented causes of dropout give no reason to expect such a reversal. We conclude that the primary result is robust to plausible departures from missing-at-random dropout.
